# Study on the Mechanism of Anti-Cerebral Ischemia-Reperfusion Injury of Ai Pian Based on Network Pharmacology and Metabolomics

**DOI:** 10.2174/0113892002403752251015105027

**Published:** 2025-10-31

**Authors:** Jianing Lian, Yilun Ma, Dazhong Lu, Peiru Wang, Mengmeng Zhang, Taiwei Dong

**Affiliations:** 1 College of Pharmacy, Shaanxi University of Chinese Medicine, Xianyang, Shaanxi, 712046, China

**Keywords:** Ai pian, cerebral ischemia-reperfusion injury, metabolomics, network pharmacology, ischemic stroke, thymidine

## Abstract

**Objective:**

The objective of this study was to investigate the mechanism of anti-cerebral ischemia-reperfusion injury (anti-CIRI) of Ai pian by using the network pharmacology approach combined with serum metabolomics technique based on UPLC-MS.

**Methods:**

The cerebral ischemia-reperfusion injury (CIRI) model was established by middle cerebral artery occlusion (MCAO). The therapeutic effect of Ai pian on CIRI rats was evaluated by behavioral test, 2,3,5-triphenyltetrazolium chloride (TTC) staining, Nissl staining, and hematoxylin-eosin (HE) staining. The active compound–potential target–disease network for Ai Pian in the treatment of CIRI was established using network pharmacology methods. Rat serum was detected by the metabolomics technique based on UPLC-MS. A Western blot was used to validate common targets of the network pharmacology approach combined with serum metabolomics.

**Results:**

The process of treating CIRI with Ai Pian involved regulating enzyme, nuclear receptor, and transcription factor activity, managing the inflammatory response, and participating in biofilm composition. Twenty endogenous potential biomarkers were screened and submitted to MetaboAnalyst 6.0 for pathway and enrichment analysis. Four metabolic pathways were identified: butanoate metabolism, fructose and mannose metabolism, alanine, aspartate, and glutamate metabolism, and pyrimidine metabolism. Fructose and mannose metabolism and pyrimidine metabolism were two key pathways. Western blot analysis suggested that DHODH, TYMS, and AKR1B1 may be targets through which therapeutic effects are exerted.

**Discussion:**

The present study made preliminary predictions on the possible mechanisms of Ai Pian against CIRI. Differential metabolites were screened and identified, and the relevant metabolic pathways potentially affected by Ai Pian were discovered to understand the importance of these markers in health and disease. However, there were also some limitations, further exploration of the molecular mechanisms at the transcriptional level was necessary to make the experimental results more reliable.

**Conclusion:**

This research contributed to the development of Ai pian as an adjunctive drug for treating CIRI and provided a basis for further research on CIRI.

## 1 INTRODUCTION

Brain apoplexy is a type of cerebrovascular disease, which is accompanied by a high incidence of morbidity, disability, and mortality, resulting in at least 6,671,000 deaths and a number of patients with disabilities each year [1]. With the acceleration of population ageing and urbanization, the prevalence trend of stroke risk factors is obvious, and the burden of disease is increasing [2]. Ischemic stroke is a typical presentation of stroke, accounting for about 80% of all strokes [3]. It is a modern clinical syndrome caused primarily by an interruption in cerebral blood flow, which induces ischemia and hypoxic necrosis of brain tissue and severe neural injuries [4]. The most effective treatment for ischemic stroke is rapidly restoring blood flow to the affected area of brain tissue at an early stage [5]. However, in some cases, it may exacerbate the damage, called CIRI [6]. Therefore, reducing CIRI is the key to improving patient prognosis. The pathogenesis of CIRI is complex, involving apoptosis, blood-brain barrier (BBB) disruption, calcium overload, oxidative stress, inflammation, energy metabolism impairment, and glutamate/neurotoxin release, among other factors [7, 8]. Currently, emerging ther apeutic approaches to CIRI are targeting immune activation and suppressing the inflammatory response [9]. The existing drugs for treating stroke include nimodipine, edaravone, *etc*. However, nimodipine dilates blood vessels, and long-term use may lead to decreased blood pressure, thereby increasing the burden on the kidneys. Side effects of edaravone include impaired liver function and abnormal kidney function [10]. Therefore, it is necessary to search for therapeutic agents that are effective and have fewer side effects.

Ai Pian is the name of a traditional Chinese medicine. The crystal is extracted and processed from fresh leaves of *Blumea balsamifera (L.) DC.*, a plant of the Asteraceae family. Ai pian is a traditional Chinese medicine used to treat stroke, with its main ingredient being the bicyclic monoterpenoid L-borneolum, derived from borneol [11]. The drug has the effect of opening the orifices, clearing the mind, removing heat, and relieving pain [12]. Modern research has shown that Ai pian possesses anti-inflammatory, pain-relieving, anti-myocardial ischemia, and brain-protective properties. Ai pian is clinically used to treat fever, spasms and convulsions, stroke, phlegm-induced fainting, throat soreness, and other related ailments. It can induce drugs into the meridian to increase their bioavailability for cardiovascular and cerebrovascular diseases [13].

Its main component, borneolum, has a bidirectional regulatory effect on the central nervous system. Borneolum can enhance the hypnotic effect of pentobarbital sodium within the dose range of 0.5–2.0 mg/kg; however, it produces central excitatory effects beyond 3.5 mg/kg. This dose-dependent characteristic is related to the differential activation of GABA receptor subtypes. Cell experiments have shown that borneolum can penetrate the blood-brain barrier (BBB), and its concentration in brain tissue can reach 6–8 times that of plasma. This characteristic provides a theoretical basis for its treatment of neurological diseases [14]. Therefore, the research group has conducted preliminary studies on Ai Pian. Previous studies by the research group have shown that prophylactic administration of Ai Pian for 3 days (0.2 g/kg/day) can improve neuronal cell impairment in the CIRI rat model and inhibit the modeling-induced increase in body temperature.

The present study made preliminary predictions on the possible mechanisms of Ai Pian against CIRI using network pharmacology. Serum from CIRI rats before and after Ai Pian intervention was analyzed by LC-MS technology. Differential metabolites were screened and identified, and the relevant metabolic pathways potentially affected by Ai Pian were discovered to understand the importance of these markers in health and disease. The experimental design was divided into the following parts: network pharmacology, animal experiments, serum metabolomics data analysis, compound-reaction-enzyme-gene network construction, and mechanism verification.

## 2 MATERIALS AND METHODS

### 2.1 Screening of Candidate Therapeutic Targets of Ai pian

Due to the relatively singular component of Ai pian, the prediction was made for the anti-CIRI mechanism of its main component (L-borneolum) using the network pharmacology approach. The chemical structure of L-borneolum was acquired from the PubChem database and saved in Standard Database Format (SDF). The potential targets of L-borneolum were collected through the PharmMapper database to establish the compound-target dataset [15].

### 2.2 Screening of Disease-relevant and Intersection Targets Between the Compound and Disease

In the GeneCards and OMIM databases, the targets of CIRI were obtained by searching the keywords “cerebral ischemic stroke” and “cerebral infarction.” The associated data were extracted and integrated to establish the disease-target dataset. Venn diagrams were generated using the Bioinformatics server to screen for intersecting targets between L-borneolum and CIRI.

### 2.3 Construction of Protein-protein Interaction (PPI) Networks

The intersection targets of drugs and diseases were uploaded into the STRING online platform, and species information was set to “Homo sapiens” to construct the PPI network. The TSV format file was then imported into Cytoscape 3.9.1 to visualize the PPI network along with the correlation magnitude.

### 2.4 Construction of the Active Ingredient-candidate Targets-diseases Network

Cytoscape 3.9.1 was used to construct the L-borneolum candidate target-cerebral ischemia-reperfusion injury network. The nodes represented the active ingredient, intersection targets, or diseases, while the edges indicated the interaction between the active ingredient and the intersection targets, or the interaction between the diseases and the intersection targets.

### 2.5 GO and KEGG Enrichment Analysis

The candidate targets of L-borneolum for treating the CIRI were uploaded into the DAVID database. Based on *P* < 0.01, the top 10 significantly enriched GO items were retained. The relevant signaling pathways enriched in the potential targets were screened by P-value to obtain the top 20 pathways.

### 2.6 Drugs and Reagents

Ai Pian (GuiZhou Miaoyao Biotech Co., Ltd.) contains approximately 90% L-borneolum according to GC-MS analysis, exceeding the 85% requirement specified in the Chinese Pharmacopoeia 2020 Edition; 2,3,5-triphenyltetrazolium chloride (TTC, Shanghai yuanye Bio-Technology Co., Ltd.); phosphate-buffered saline (PBS, Boster Biological Technology Co., Ltd.); 4% paraformaldehyde fixative (Boster Biological Technology Co., Ltd.); methanol, acetonitrile, formic acid, and isopropyl alcohol (Chromatography grade, Fisher Chemical).

### 2.7 Experimental Animals

SPF male SD rats, aged 6-8 weeks old, 250±20 g, were obtained by Chengdu Dashuo Biotechnology Co., Ltd. (Certificate of Conformity No.: SCXK-Chuan, 2020-0030). The room temperature was 20~25 °C. The humidity was 40~60%. All animal experiments were designed on the basis of the related regulations of Shaanxi University of Chinese Medicine’s experimental animal ethics committee (SUCMDL2021 0309006).

### 2.8 Grouping and Administration of Experimental Animals

About 6 g of Ai pian powder was accurately weighed and ground evenly with 3 mL of 1% polysorbate 80 in a mortar and pestle. Then, 297 mL of pure water was added in small amounts multiple times to make a 0.02 g/mL solution. The solution was stored in a refrigerator at 4°C (pre-warm at 37°C and shake well before each use).

After 1 week of acclimatization, 18 male SD rats were randomly divided into 3 groups (n = 6 each) based on body weight: the sham operation group, model group, and Ai Pian group. The Ai Pian group was administered the Ai Pian solution by gavage at a dose of 10 mL/kg. Except for the model group, the other two groups were given the same volume of saline. Dosing was performed once daily for 3 consecutive days. Thirty minutes after the third prophylactic administration, rats in the model group and the Ai Pian group underwent CIRI model induction. Rats in the sham operation group did not undergo the procedure of inserting sutures from the common carotid artery (CCA) into the internal carotid artery (ICA); all other procedures were the same as in the other two groups. At 24 hours after successful modeling, the corresponding drugs were again administered to each group of rats by gavage at the same dosage. Thirty minutes after the last administration, the rats were euthanized to obtain complete brain tissue and serum for subsequent experiments.

### 2.9 Animal Models Preparation

Each animal was anesthetized, and the skin and subcutaneous tissue were incised to expose the right common carotid artery (CCA), external carotid artery (ECA), and internal carotid artery (ICA), followed by ligation of the proximal ends of the CCA and ECA [16]. The ICA was temporarily occluded with an arterial clamp. Approximately 2 mm from the bifurcation of the CCA, a slip knot was tied with thin thread, and a small incision was made about 1 mm below the knot to insert the suture from the CCA into the ICA. The slip knot was then tied firmly to fix the suture, and the wound was cleaned and sutured [5]. After 2 hours of ischemia, the suture was withdrawn 1 cm outward, and the excess part was cut off to allow reperfusion. The sham operation group underwent the same procedure except that the suture was not inserted from the CCA into the ICA. The Zea-Longa score was used to assess neurological function in a blinded manner once all animals were fully awake [17].

### 2.10 Behavioral Test

Neurological deficits were assessed using the Zea-Longa score [18]. The scoring criteria are as follows (Table **1**) [19]. A higher score indicates more severe neurological deficits. Rats with a Zea-Longa score ≥1 were selected for subsequent experiments.

### 2.11 TTC Staining

Brain tissues were quickly removed and frozen at –20 °C for 20 minutes. The brain tissues were then sliced into five 2-mm sections along the coronal plane on ice. The slices were immediately transferred to 2% TTC solution and incubated at 37 °C for 30 minutes in the dark. Afterwards, the slices were immersed in 4% paraformaldehyde for 24 hours and then photographed. ImageJ software was used to measure the cerebral infarction area and calculate the percentage of the infarcted area.

### 2.12 Histopathological Observation

Brain tissues were quickly removed, fixed in 4% paraformaldehyde for 24 h, and dehydrated and embedded in paraffin. Slices of 4 μm in thickness were prepared to stain with HE and Nissl stain solution. Pathological histological features in each group were viewed under a light microscope.

### 2.13 LC-MS Analysis

About 100 µL serum samples were extracted with 300 µL methanol:acetonitrile (1:1, v/v) solution. Extracted metabolites were centrifuged for 10 min at 13,000 g at 4°C, and the supernatant was transferred to sample vials for LC-MS analysis.

#### 2.13.1 Chromatographic Conditions (UHPLC-Exploris 240 System)

Chromatographic column: HSS T3 column (100 mm × 2.1 mm i.d., 1.8 μm; Waters, Milford, USA) was used. The mobile phases comprised 0.1% formic acid in water: acetonitrile (95:5, v/v) (solvent A) and 0.1% formic acid in acetonitrile:isopropanol: water (47.5:47.5:5, v/v) (solvent B). The gradient elution conditions are shown in Table **2**.

#### 2.13.2 MS Conditions

The mass spectrometric data were obtained using the UHPLC-Exploris 240 Mass Spectrometer. The specific parameters are shown in Table **3**.

### 2.14 Multivariate Statistical Analysis and Identification of Potential Biomarkers

Principal component analysis (PCA) was performed for unsupervised statistical analysis. Samples were grouped and analyzed according to the score plot obtained from supervised partial least squares discriminant analysis (PLS-DA), and 200 random permutations were used to assess the model and avoid overfitting. Metabolites with a variable importance in projection (VIP) > 1 and a *p*-value < 0.05 in Student’s t-test were considered significantly different. T-tests on the peak intensities of these metabolites were conducted using SPSS 26.0 software to compare biomarker changes between test groups. Biomarker identification and pathway enrichment analysis were performed using the KEGG database, MetaboAnalyst 6.0 platform, and Bioinformatic server, with *p* < 0.05 defined as the significance threshold.

### 2.15 Integrated Analysis of Metabolomics and Network Pharmacology

Differential metabolites and intersecting targets were imported into Cytoscape 3.9.1 The Metscape plug-in was used to construct a compound-reaction-enzyme-gene network to visualise the metabolic pathways.

### 2.16 Western Blot Analysis

The total protein was extracted from brain tissue using ice-cold RIPA lysis buffer. The protein content was determined using the BCA protein quantification kit. Protein samples were then prepared through heating denaturation and subsequently stored at -20°C. After electrophoresis, the samples were transferred to PVDF and sealed in 5% skim milk. Primary antibodies, including beta-actin (1:5000), TYMS (1:1000), AKR1B1 (1:5000), DHODH (1:1000), and the appropriate secondary antibody, were added and incubated. Finally, detection was performed using a chemiluminescence system.

### 2.17 Statistical Analysis

The data were presented as the mean ± standard deviation (SD). The illustrations were created using GraphPad Prism 8.0 software. The t-test was used for statistical differences between two groups by SPSS 26.0. *p* < 0.05 was set as the significance threshold.

## 3 RESULTS

### 3.1 Candidate Therapeutic Targets of Ai pian

In the PharmMapper database, 124 targets were filtered as Candidate therapeutic targets of Ai pian.

### 3.2 Disease-related Targets and Intersection Targets Between the Compound and Disease

**Fig. (1):**
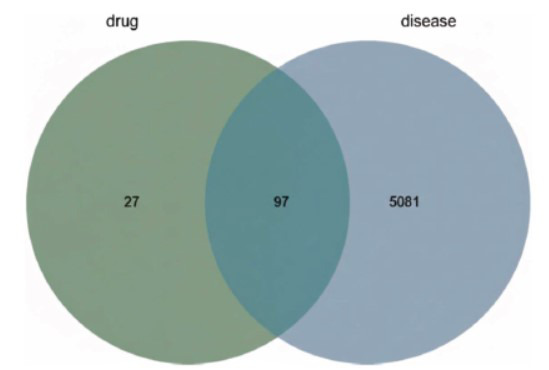
Venn diagram of the intersection targets of Ai pian and CIRI.

A total of 5178 disease-related targets were filtered after merging and de-duplication by searching the Genecards and OMIM databases using “cerebral ischemic stroke” and “cerebral infarction” as keywords. According to the Venn diagram, 97 common targets were obtained (Fig. **1**).

### 3.3 PPI Network and Core Targets Screening

**Fig. (2):**
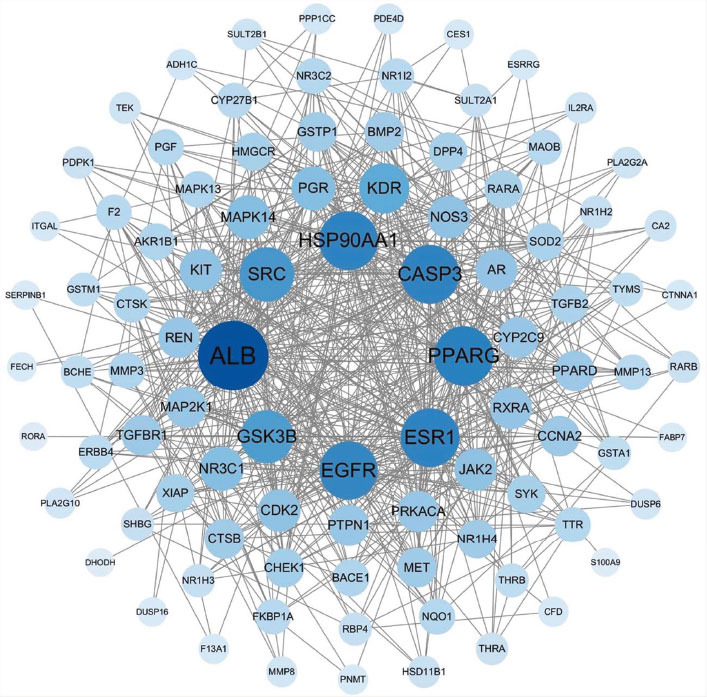
PPI network of Ai pian in the treatment of CIRI. The size and color of the nodes indicate the different degrees.

The 97 intersection targets of the drug and disease were imported into the STRING database, and the results were uploaded into Cytoscape 3.9.1 for visualization (Fig. **2**). The top 10 key proteins identified were ALB, PPARG, CASP3, ESR1, HSP90AA1, EGFR, SRC, GSK3B, KDR, and PGR. The topological parameters are shown in Table **4**.

### 3.4 Active Compound-potential Target-disease Network

The L-borneolum-potential target-cerebral ischemia-reperfusion injury network is shown in Fig. (**3**), with a total of 99 nodes and 194 edges. The red oval represents CIRI, the purple octagon represents Ai pian, and the yellow V-shape represents the potential targets of Ai pian in protecting against CIRI.

### 3.5 GO and KEGG Enrichment Analysis

**Fig. (4):**
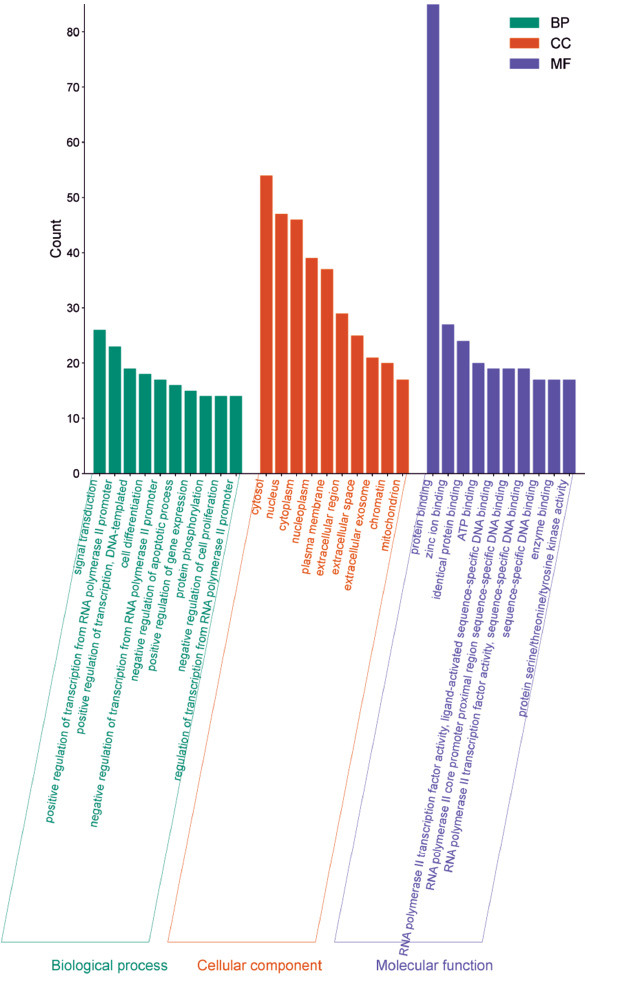
GO enrichment analysis. Green, orange, and purple represent the enrichment analysis of biological process, cellular component, and molecular function, respectively.

**Fig. (5):**
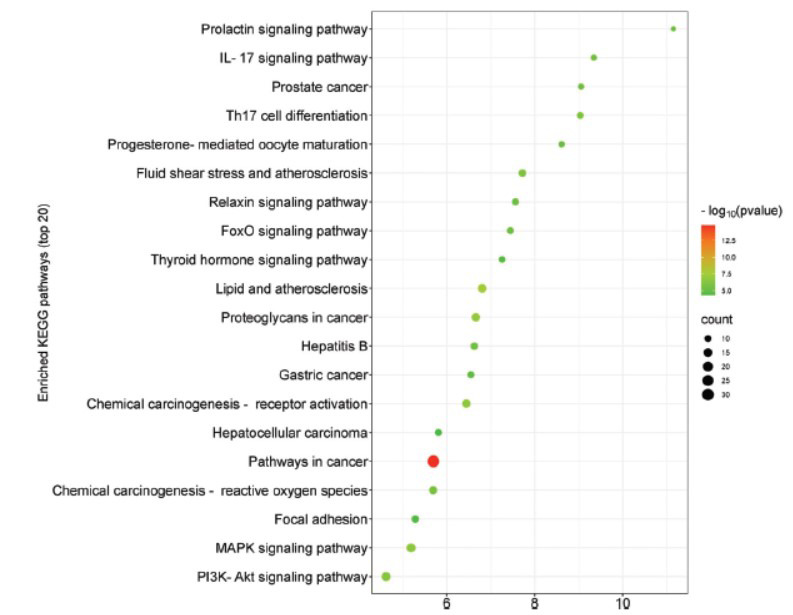
Bubble diagram of the top 20 pathways based on KEGG enrichment analysis.

There were 331 entries related to biological processes, mainly involved in signal transduction, positive regulation of transcription from the RNA polymerase II promoter, and positive regulation of DNA-templated transcription. There were 45 entries related to cellular components, which mainly included the cytosol, nucleus, and cytoplasm. There were 89 entries related to molecular functions, mainly associated with protein binding, zinc ion binding, identical protein binding, ATP binding, RNA polymerase II transcription factor activity, and ligand-activated sequence-specific DNA binding (Fig. **4**). The mechanism of Ai pian against CIRI was primarily related to the MAPK signaling pathway, PI3K-Akt signaling pathway, IL-17 signaling pathway, and FoxO signaling pathway (Fig. **5**).

### 3.6 Ai pian Attenuated CIRI in Rats

**Fig. (6):**
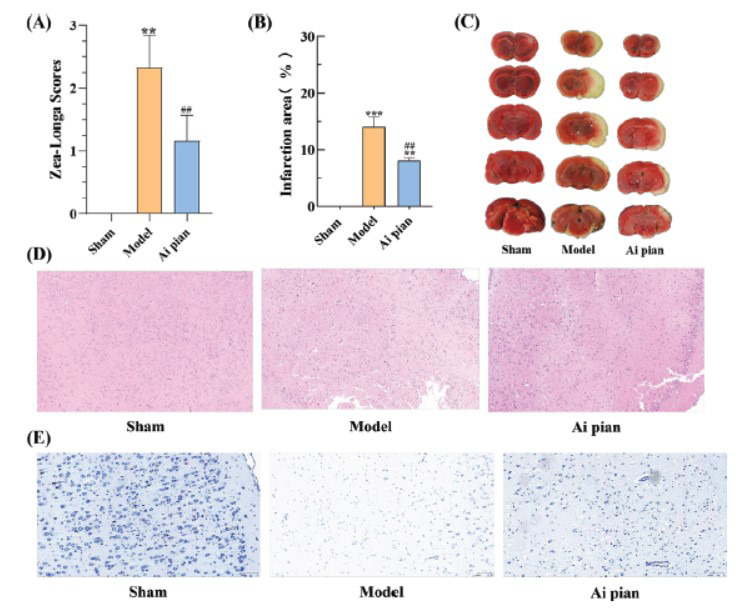
Ai pian protects against CIRI in rats. (**A**) Zea-Longa score (n = 6); (**B**, **C**) Statistical analysis of TTC staining of cerebral infarction area and infarction area (n = 3); (**D**) HE staining (scale bars: 300 µm; n = 3); (**E**) Nissl staining (scale bars: 100 µm; n = 3). The data were presented as the means ± SD. **p < 0.01 *vs*. Sham; ***p < 0.001 *vs*. Sham; ##*p* < 0.01 *vs*. Model.

Zea-Longa score results indicated that neurological function was normal in the sham operation group. Compared with the sham operation group, the Zea-Longa score in the model group was significantly increased (*P* < 0.01), suggesting that the neurological function was severely impaired. However, compared with the model group, the Zea-Longa score in the Ai pian group was significantly decreased (*P* < 0.01). Ai pian could repair neurological dysfunction (Fig. **6A**).

All brain slices of the sham operation group showed a red color with no infarction, while those of the model group showed a white color with 14% infarction. Compared with the model group, the percentage of infarcted area decreased to 8% in the Ai pian group, with a statistically significant difference (*p* < 0.01; Fig. **6B** and **6C**). It indicated that Ai pian treatment significantly ameliorated the cerebral infarction area.

Additionally, the pathological features of the infarction area were assessed using HE staining (Fig. **6D**) and Nissl staining (Fig. **6E**). Neurons in the sham operation group appeared normal and were arranged in a regular pattern, whereas in the model group, neurons showed swelling, nuclear atrophy, and nuclear condensation, with a loose and irregular arrangement. Ai pian treatment significantly improved neuronal damage. In summary, Ai pian treatment effectively attenuated CIRI in rats.

### 3.7 Analysis of the Rats' Serum Metabolomics Data

**Fig. (7):**
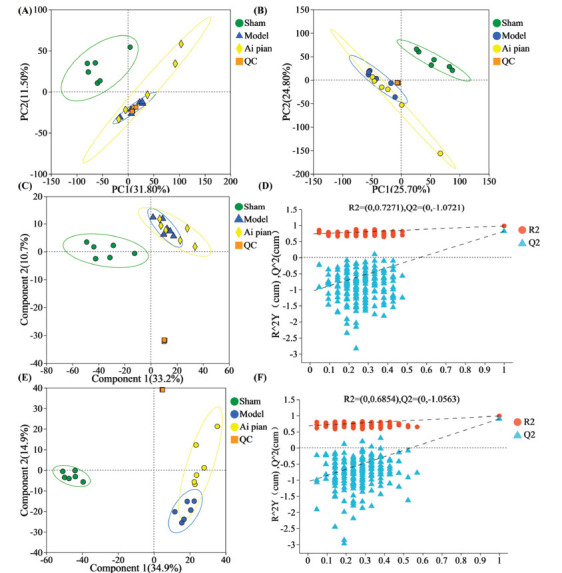
Multivariate statistical analysis of rats' serum metabolomics. (**A**) PCA score plot in positive ion mode; (**B**) PCA score plot in negative ion mode; (**C**) PLS-DA score plot in positive ion mode; (**D**) Permutation tests in positive ion mode; (**E**) PLS-DA score plot in negative ion mode; (**F**) Permutation tests in negative ion mode; (n = 6).

According to PCA (Fig. **7A** and **7B**), the sham operation group significantly differed from the model group, indicating the CIRI model established in this study was reliable. Each group exhibited clustering, especially QC samples, indicating the high stability of LC-MS detection during the experiment system.

The differential serum endogenous metabolites were further visualized using a supervised PLS-DA model. According to PLS-DA (Fig. **7C**-**E**), R^2^Y = 0.727 and Q^2^ = 1.072 in the positive ion mode, and R^2^Y = 0.685 and Q^2^ = 1.056 in the negative ion mode. The results showed that the three groups of samples exhibited clustering and significant separation. The model established in this study was reliable and had good predictive ability. Additionally, 200 permutation tests were performed to validate possible overfitting of the PLS-DA model. The intercept of Q^2^ on the y-axis was negative (Fig. **7D** and **F**), indicating that the model did not overfit and that the data were authentic and trustworthy.

### 3.8 Identification of Endogenous Potential Biomarkers

As shown in Fig. (**8A**-**D**), the total number of serum metabolites with significant differences between the sham operation group and the model group was 709, of which 345 were upregulated and 364 were downregulated. The total number of serum metabolites with significant differences between the Ai pian group and the model group was 298, with 170 upregulated and 128 downregulated ones. There were 121 metabolites common to both comparisons. By searching and matching the secondary mass spectrometry information of differential metabolites that met the screening criteria with the HMDB online database, 20 significantly different metabolites were identified and screened (Table **5**).

**Fig. (10):**
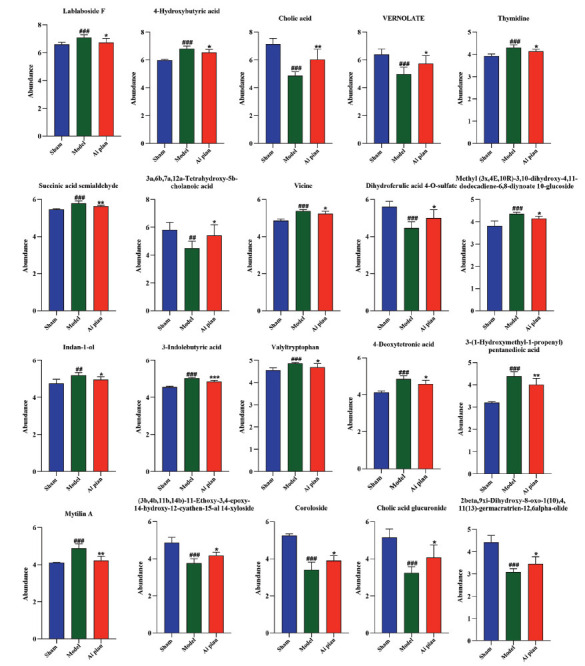
Impact values of differential metabolites (Comparison with sham operation group: “#” means 0.01 ≤ *p* < 0.05, “##” means 0.001 ≤ *p* < 0.01, “###” means *p* < 0.001; Comparison with model group: “*” means 0.01 ≤ *p* < 0.05, “**” means 0.001 ≤ *p* < 0.01; “***” means *p* < 0.001).

The 20 significantly different metabolites were analyzed by hierarchical cluster analysis to obtain the heat map. As shown in Fig. (**9**), each row represented the differential metabolites from different samples. Each column represented the expression level of all differential metabolites in each sample. The heat map visually depicted the content variations of 20 significantly different metabolites between groups. The significantly different metabolites in the sham operation group and model group can be readily separated. The content level of the differential metabolites in the Ai pian group had a trend of callback to the sham operation group. The statistical analysis results indicated that the level of significantly different metabolites in the Ai pian group was closer to those in the sham operation group than those in the model group (Fig. **10**). Therefore, the 20 significantly different metabolites can be subsequently analyzed as endogenous potential biomarkers for the treatment of CIRI with Ai pian.

**Fig. (11):**
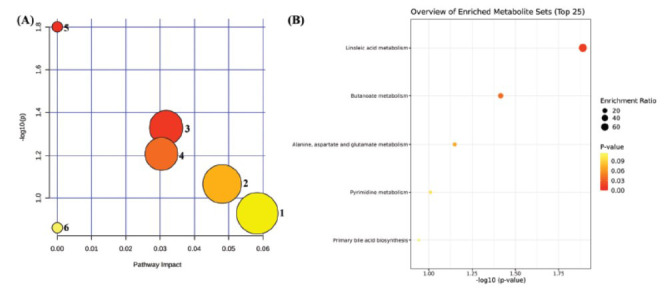
(**A**) Metabolic pathway analysis diagram (1. Pyrimidine metabolism; 2. Alanine, aspartate and glutamate metabolism; 3. Butanoate metabolism; 4. Fructose and mannose metabolism; 5. Linoleic acid metabolism; 6. Primary bile acid biosynthesis); (**B**) Metabolite enrichment analysis bubble diagram.

The 20 potential biomarkers identified were submitted to MetaboAnalyst 6.0 for pathway and enrichment analysis (Fig. **11A** and **11B**). The results indicated that the candidate biomarkers were involved in linoleic acid metabolism, butanoate metabolism, fructose and mannose metabolism, alanine, aspartate, and glutamate metabolism, pyrimidine metabolism, and primary bile acid biosynthesis pathways. Based on the impact > 0, 4 key metabolic pathways were selected: butanoate metabolism, fructose and mannose metabolism, alanine, aspartate and glutamate metabolism, and pyrimidine metabolism.

### 3.9 Compound-reaction-enzyme-gene Network

**Fig. (12):**
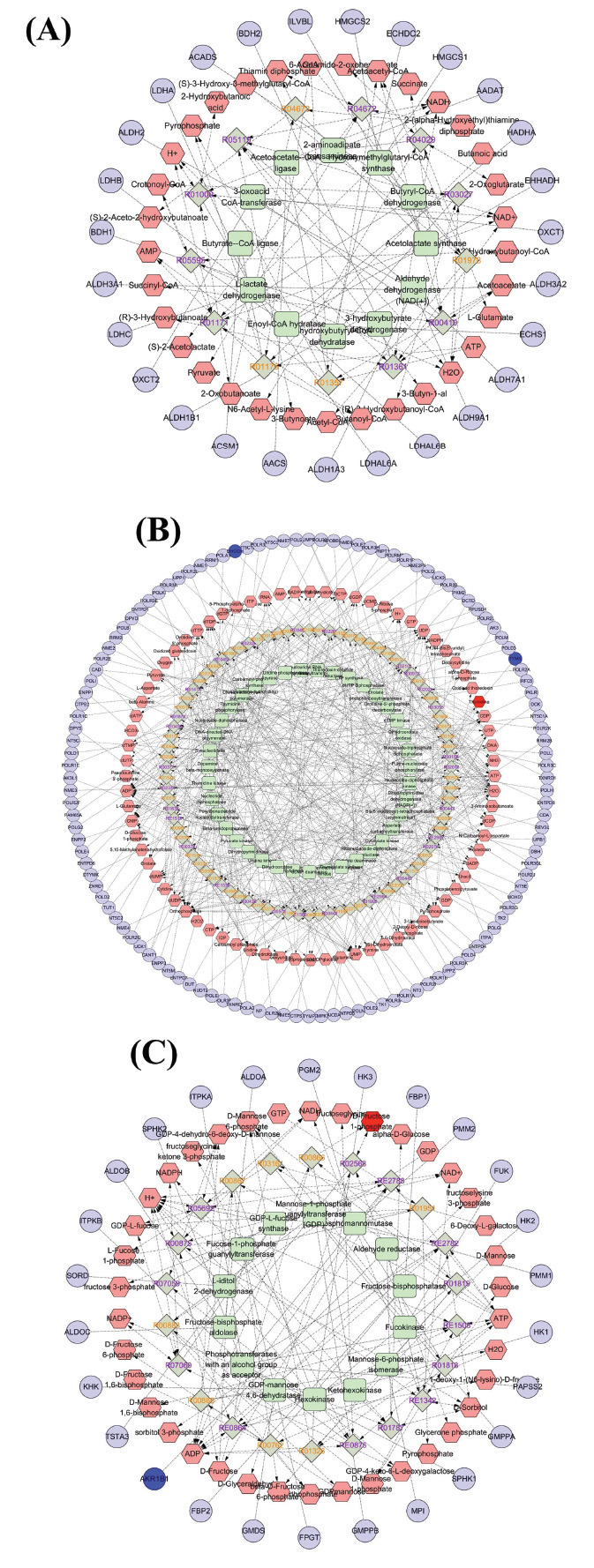
Compound-reaction-enzyme-gene network (**A**) Butanoate metabolism; (**B**) Pyrimidine metabolism; (**C**) Fructose and mannose metabolism (red hexagon, pink hexagon, blue circle, purple circle, green square, and light green diamond tables represent serum differential metabolites, other metabolites, potential gene targets, other targets, enzymes, and reactions).

The compound-reaction-enzyme-gene network diagram was constructed by integrating metabolomics with network pharmacology (Fig. **12**), focusing on the metabolic pathways of pyrimidine metabolism and fructose and mannose metabolism. The key targets in the pyrimidine metabolism pathway were DHODH and TYMS, while AKR1B1 was identified as the key target in the fructose and mannose metabolism pathway.

### 3.10 Western Blot Analysis

**Fig. (13):**
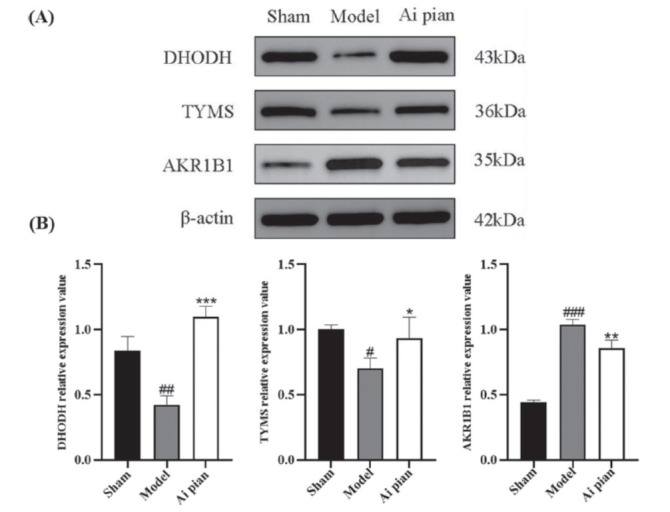
Effect of Ai pian on expression of DHODH, TYMS and AKR1B1 (**A**) Western blot images of DHODH, TYMS and AKR1B1; (**B**) Statistical analysis of the expression of DHODH, TYMS and AKR1B1 (Comparison with sham operation group: “#” means 0.01 ≤ *p* < 0.05, “##” means 0.001 ≤ *p* < 0.01, “###” means *p* < 0.001; Comparison with model group: “*” means 0.01 ≤ *p* < 0.05, “**” means 0.001 ≤ *p*.

Western blots were used to validate common targets of metabolomics and network pharmacology (Fig. **13**). The levels of DHODH and TYMS were significantly reduced (*p* ˂ 0.05), and the level of AKR1B1 was significantly increased (*p* ˂ 0.05) in rats with CIRI. However, the levels of DHODH and TYMS were significantly increased (*p* ˂ 0.05), and the level of AKR1B1 was significantly reduced (*p*˂ 0.05) in the Ai pian group. After administration of Ai pian, the expression levels of DHODH, TYMS, and AKR1B1 proteins were reversed to approach normal rats.

## 4 DISCUSSION

Ischemic stroke is a serious condition that endangers human health. Two methods of restoring cerebral blood flow after ischaemic stroke are thrombolysis and mechanical recanalisation [20]. However, restoring blood supply can lead to increased generation of reactive oxygen species; reperfusion can actually worsen the brain damage caused by ischemia in some situations [21]. CIRI is an important pathological stage in patients with Ischemic stroke, occurring after blood perfusion is restored. It is involved in various pathological processes, such as endoplasmic reticulum stress [22], apoptosis [23], inflammatory response [24], pyroptosis [25], and ferroptosis [12]. Over the past few years, modern medicine has made some progress in the diagnosis and treatment of CIRI. Anti-inflammatory and antioxidant drugs, such as butylphthalide [26] and edaravone [27], are commonly used for treating CIRI in clinical practice. However, there are still problems, such as postoperative rehabilitation difficulties and drug adverse reactions. Therefore, new treatment plans and drugs are urgently needed for treating the CIRI. Traditional Chinese medicines, which have the advantages of low side effects and multi-target and multi-pathway action, have received more and more attention from researchers.

Ai Pian is an extract of the leaves of *Blumea balsamifera*, which has a wide range of clinical applications [28]. In this study, a complete network of network pharmacology and metabolomics was established using the network pharmacology approach combined with serum metabolomics technique based on UPLC-MS to confirm the treatment effect of Ai Pian on CIRI and screen potential therapeutic targets.

According to the network pharmacology screening results, 97 potential targets of Ai pian against CIRI were obtained. The proteins of ALB, PPARG, CASP3, ESR1, HSP90AA1, EGFR, SRC, GSK3B, KDR, and PGR could play an important regulatory role in treating CIRI with Ai pian. The process of treating CIRI with Ai pian involved regulating enzyme, nuclear receptor, and transcription factor activity, as well as managing the inflammatory response and participating in the composition of the biofilm. The process of treating CIRI with Ai pian was related to 112 signaling pathways. The FoxO family of forkhead transcription factors is a subfamily within the larger Fox protein family and, in mammals, includes FOXO1 (FKHR/FOXO1a), FOXO3 (FKHRL1/FOXO3a), and FOXO4 (AFK/FOXO6). Research has shown that FoxO3a is highly expressed in the hippocampus and cortical regions of the brain and plays a crucial role in regulating multiple metabolic pathways and neuronal functions [29]. FOXO3a acts as a core regulatory factor in processes such as cellular homeostasis, oxidative stress response, apoptosis, and autophagy, and functions downstream of the PI3K/Akt signaling pathway [30]. Upon activation, Akt translocates into the nucleus and phosphorylates FoxO3a at specific residues (T32, S253, S315), which reduces its DNA-binding ability and facilitates its interaction with 14-3-3 proteins, leading to altered transcriptional activity.

FoxO3a is transported to the cytoplasm as part of a complex, which prevents its re-entry into the nucleus by retaining it in the cytoplasm. This cytoplasmic retention inhibits Bim-mediated apoptosis, thereby promoting cell survival [31, 32]. Under stress conditions, FoxO3a activity can also be suppressed through phosphorylation by MAPK [33].

Serum from CIRI rats, before and after Ai pian intervention, was analyzed using LC-MS technology. This analysis identified 20 endogenous potential biomarkers and highlighted 6 related metabolic pathways: linoleic acid metabolism, butanoate metabolism, fructose and mannose metabolism, alanine, aspartate and glutamate metabolism, pyrimidine metabolism, and primary bile acid biosynthesis. A compound-reaction-enzyme-gene network was constructed by integrating metabolomics with network pharmacology, with a focus on the metabolic pathways of pyrimidine metabolism and fructose and mannose metabolism.

Pyrimidine metabolism involves a complex network of enzymatic reactions integrating nucleoside salvage, nucleotide synthesis, and catalytic degradation of pyrimidines [34]. As a subset of nucleotide metabolism, disruptions in pyrimidine metabolism can contribute to diseases affecting the immune, circulatory, and nervous systems. ATP and GTP serve as the primary cellular energy carriers, with high-energy phosphate bonds providing the energy required for pyrimidine synthesis [35]. Thymidine acts as a DNA synthesis inhibitor by blocking the cell cycle at the G1/S phase, prior to DNA replication. Thymidylate synthase (TYMS) is a key enzyme essential for DNA biosynthesis and repair [36]. Dihydroorotate dehydrogenase (DHODH) catalyzes the fourth step in de novo pyrimidine synthesis, and its inhibition disrupts DNA synthesis and cell cycle progression [37].

Disorders in fructose and mannose metabolism indicate abnormal energy metabolism in CIRI rats. Glucose circulates freely in the blood and, upon entering cells, is phosphorylated by ATP to form glucose-6-phosphate (G-6-P), a critical intermediate in glucose metabolism. Glucose also serves as a vital energy source for cells [38].

AKR1B1 is a key enzyme in the glucose metabolism pathway and is implicated in the neuronal damage process of stroke [39]. In the present study, the thymidine content of the model group rats was abnormal, while the Ai pian group showed a trend of returning to the levels of the sham operation group. Western blot suggested that DHODH, TYMS, and AKR1B1 may be targets through which therapeutic effects are exerted. Therefore, it is speculated that Ai pian may regulate pyrimidine metabolism, fructose and mannose metabolism by controlling DHODH, TYMS, and AKR1B1, playing a role in preventing and treating CIRI.

## STUDY LIMITATIONS

This study has some limitations. Firstly, there are inherent limitations in predicting targets solely using databases. Network pharmacology, which requires continuous improvement and refinement, has certain shortcomings. Secondly, this study only conducted protein-level detection on the common targets identified by network pharmacology and metabolomics. Since the process of translating mRNA into protein is influenced by various factors, the mechanism validation is insufficient. Therefore, further exploration of the molecular mechanisms at the transcriptional level is necessary to make the experimental results more reliable.

## CONCLUSION

Twenty endogenous potential biomarkers were screened and identified, indicating that CIRI causes abnormalities in four metabolic pathways. Fructose and mannose metabolism and pyrimidine metabolism were identified as two key pathways through compound-reaction-enzyme-gene network analysis. DHODH, TYMS, and AKR1B1 were identified as key targets for Ai pian’s anti-CIRI effects. A comprehensive network integrating network pharmacology and metabolomics was established using UPLC-MS–based serum metabolomics combined with network pharmacology to explore the biological processes by which Ai pian regulates metabolic pathways to combat CIRI. Moreover, this research contributes to the development of Ai pian as an adjunctive therapy for treating CIRI.

## Figures and Tables

**Fig. (3):**
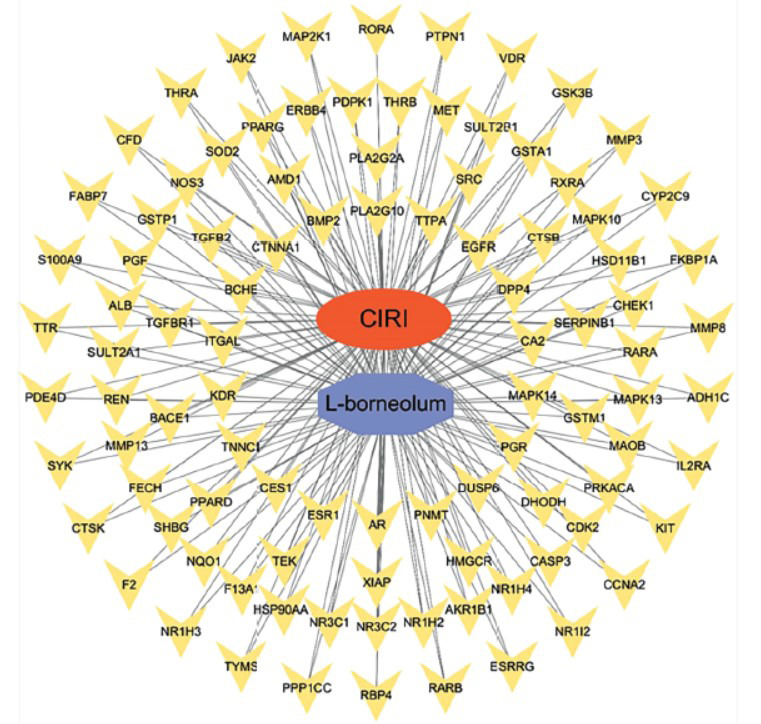
L-borneolum-potential target-cerebral ischemia-reperfusion injury network diagram.

**Fig. (8):**
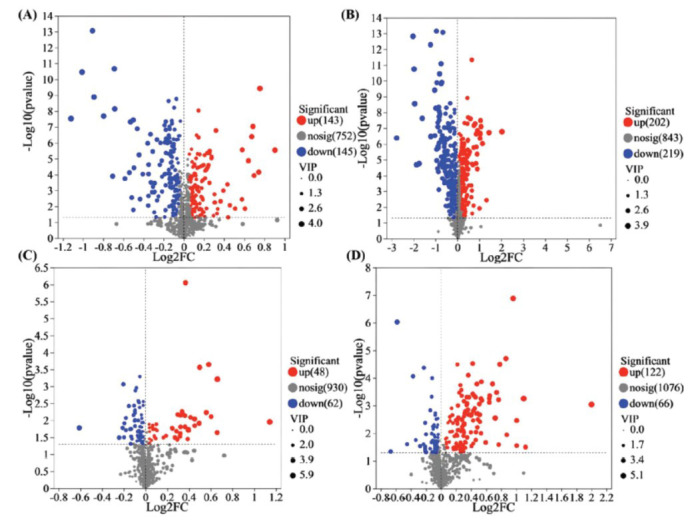
VIP plot obtained by the OPLS-DA model. (**A**) Sham *vs*. Model groups in positive ion mode; (**B**) Sham *vs*. Model groups in negative ion mode; (**C**) Ai pian *vs*. Model groups in positive ion mode; (**D**) Ai pian vs. Model groups in negative ion mode.

**Fig. (9):**
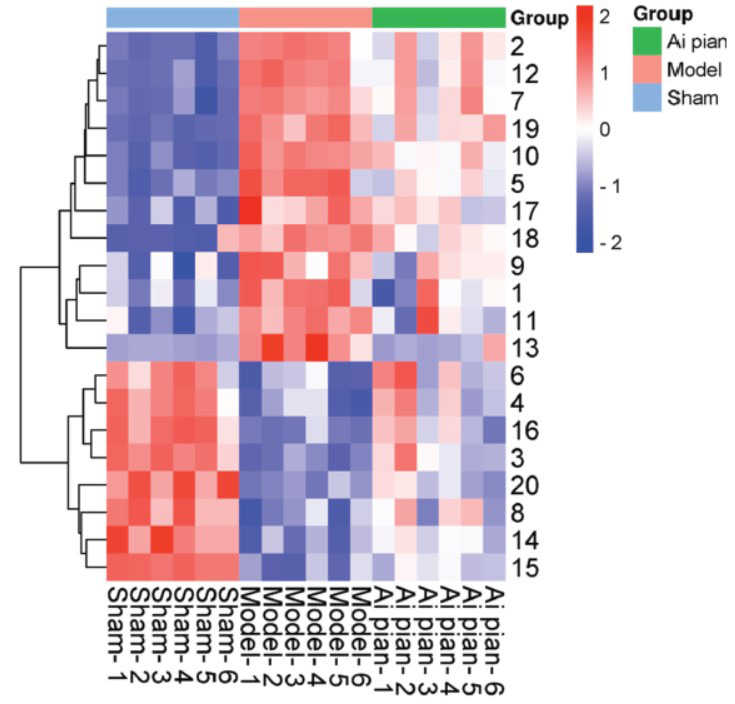
Visualization heat map of differential metabolites (The numbers on the right are the serial numbers of metabolites in Table **3**).

**Table 1:** Zea-Longa score.

**Rat Behavior**	**Score**
No symptom of neurological impairment	0 point
Rats could not fully extend the contralateral forelimb	1 point
The body turned to the hemiplegic side while walking	2 points
The body leaned to the hemiplegic side when walking	3 points
No autonomous activity accompanied by cognitive impairment	4 points

**Table 2:** The gradient elution conditions.

**Time**	**Mobile Phases**	**Sample Injection Volume**	**Flow Rate**	**Column Temperature**
0 to 0.1 min	0% B to 5% B	3 µL	0.40 mL/min	40°C
0.1 to 2 min	5% B to 25% B			
2 to 9 min	25% B to 100% B			
9 to 13 min	100% B to 100% B			
13 to 13.1 min	100% B to 0% B			
13.1 to 16 min	0% B to 0% B			

**Table 3:** The MS parameters.

**Description**	**Parameters**
Scan type (m/z)	70-1050
Sheath gas flow rate (arb)	60
Aux gas flow rate (arb)	20
Heater temp (°C)	350
Capillary temp (°C)	320
Spray voltage (+) (V)	3400
Spray voltage (-) (V)	-3000
S-Lens RF Level	70
Normalized collision energy (eV)	20, 40, 60
Resolution (Full MS)	60000
Resolution (MS^2^)	15000

**Table 4:** The topological parameters of the top 10 key proteins.

**Target**	**Closeness Centrality**	**Betweenness Centrality**	**Degree**
ALB	0.0078125	1585.250642	59
PPARG	0.006944444	732.6655075	44
CASP3	0.006944444	457.1691683	43
ESR1	0.006896552	701.1450369	43
HSP90AA1	0.006944444	649.2819608	43
EGFR	0.006896552	624.5513258	42
SRC	0.006535948	536.1818477	37
GSK3B	0.006493506	323.1828647	35
KDR	0.006289308	291.8760109	31
PGR	0.00591716	87.00801523	22

**Table 5:** Information on serum differential metabolites.

**Number**	**Name**	**HMDB**	**Formula**	**Rt/min**	**M/Z**	**VIP Value**	***P* Value**	**Sham/Model**	**Model /Ai pian**
1	Lablaboside F	HMDB0031961	C_66_H_106_O_31_	4.6954	696.33	1.1967	8.33E-04	up	down
2	4-Hydroxybutyric acid	HMDB0000710	C_4_H_8_O_3_	0.9124	103.04	1.7251	8.99E-07	up	down
3	Cholic acid	HMDB0000619	C_24_H_40_O_5_	6.2845	407.28	2.8659	4.61E-07	down	up
4	VERNOLATE	HMDB0004702	C_10_H_21_NOS	6.2049	451.27	2.1258	2.16E-04	down	up
5	Succinic acid semialdehyde	HMDB0001259	C_4_H_6_O_3_	0.8510	101.02	1.0610	6.11E-05	up	down
6	3a,6b,7a,12a-Tetrahydroxy-5b-cholanoic acid	HMDB0000399	C_24_H_40_O_6_	6.1729	405.26	1.9796	1.38E-03	down	up
7	Vicine	HMDB0034073	C_10_H_16_N_4_O_7_	1.8438	269.09	1.7637	4.51E-07	up	down
8	Dihydroferulic acid 4-O-sulfate	HMDB0041724	C_10_H_12_O_7_S	3.9684	275.02	1.9431	8.98E-05	down	up
9	Indan-1-ol	HMDB0059601	C_9_H_10_O	0.6726	401.22	1.1040	2.59E-03	up	down
10	3-Indolebutyric acid	HMDB0002096	C_12_H_13_NO_2_	6.4554	424.22	1.7097	9.10E-09	up	down
11	Valyltryptophan	HMDB0029138	C_16_H_21_N_3_O_3_	3.4107	336.19	1.3193	1.17E-04	up	down
12	4-Deoxytetronic acid	HMDB0000498	C_4_H_6_O_2_	1.8545	87.04	2.0569	4.33E-06	up	down
13	Mytilin A	HMDB0039002	C_13_H_20_N_2_O_8_	6.0638	374.15	2.1073	2.49E-05	up	down
14	(3b,4b,11b,14b)-11-Ethoxy-3,4-epoxy-14-hydroxy-12-cyathen-15-al 14-xyloside	HMDB0034617	C_27_H_42_O_8_	6.2898	539.29	1.9260	2.74E-05	down	up
15	Coroloside	HMDB0033703	C_35_H_54_O_12_	6.8703	687.33	2.5461	8.45E-07	down	up
16	Cholic acid glucuronide	HMDB0002577	C_30_H_48_O_11_	6.0560	583.31	2.5570	1.02E-05	down	up
17	Thymidine	HMDB0000273	C_10_H_14_N_2_O_5_	2.0246	287.09	1.0791	2.12E-04	up	down
18	Methyl(3x,4E,10R)-3,10-dihydroxy-4,11 dodecadiene-6,8-diynoate 10-glucoside	HMDB0040897	C_19_H_24_O_9_	0.6475	438.18	1.7066	2.20E-04	up	down
19	3-(1-Hydroxymethyl-1-propenyl)pentanedioic acid	HMDB0033092	C_9_H_14_O_5_	3.6912	247.08	2.0620	5.08E-08	up	down
20	2beta,9xi-Dihydroxy-8-oxo-1(10),4,11(13)-germacratrien-12,6alpha-olide	HMDB0036662	C_15_H_18_O_5_	6.1037	277.11	2.1491	2.81E-06	down	up

## Data Availability

All data generated or analyzed during this study are included in this published article.

## LIST OF ABBREVIATIONS

CIRI: Cerebral Ischemia-reperfusion Injury
MCAO: Middle Cerebral Artery Occlusion
BBB: Blood-brain Barrier
SDF: Standard Database Format
CCA: Common carotid artery
ECA: External Carotid Artery
ICA: Internal Carotid Artery
PCA: Principal Component Analysis
